# The Production of ACC Deaminase and Trehalose by the Plant Growth Promoting Bacterium *Pseudomonas* sp. UW4 Synergistically Protect Tomato Plants Against Salt Stress

**DOI:** 10.3389/fmicb.2019.01392

**Published:** 2019-06-19

**Authors:** Ma. del Carmen Orozco-Mosqueda, Jin Duan, Mercedes DiBernardo, Elizabeth Zetter, Jesús Campos-García, Bernard R. Glick, Gustavo Santoyo

**Affiliations:** ^1^Department of Biology, University of Waterloo, Waterloo, ON, Canada; ^2^Facultad de Agrobiología “Presidente Juárez,” Universidad Michoacana de San Nicolás de Hidalgo, Uruapan, Mexico; ^3^Instituto de Investigaciones Químico Biológicas, Universidad Michoacana de San Nicolás de Hidalgo, Morelia, Mexico

**Keywords:** plant growth-promoting bacteria, saline soils, ACC deaminase, trehalose synthase, ethylene

## Abstract

Soil salinity is a major problem in agriculture. However, crop growth and productivity can be improved by the inoculation of plants with beneficial bacteria that promote plant growth under stress conditions such as high salinity. Here, we evaluated 1-aminocyclopropane-1-carboxylate (ACC) deaminase activity and trehalose accumulation of the plant growth promoting bacterium *Pseudomonas* sp. UW4. Mutant strains (mutated at *acdS, treS*, or both) and a trehalose over-expressing strain (*OxtreS*) were constructed. The *acdS* mutant was ACC deaminase minus; the *treS^-^* strain significantly decreased its accumulation of trehalose, and the double mutant was affected in both characteristics. The *OxtreS* strain accumulated more trehalose than the wild-type strain UW4. Inoculating tomato plants subjected to salt stress with these strains significantly impacted root and shoot length, total dry weight, and chlorophyll content. The evaluated parameters in the single *acdS* and *treS* mutants were impaired. The double *acdS*/*treS* mutant was negatively affected to a greater extent than the single-gene mutants, suggesting a synergistic action of these activities in the protection of plants against salt stress. Finally, the *OxtreS* overproducing strain protected tomato plants to a greater extent under stress conditions than the wild-type strain. Taken together, these results are consistent with the synergistic action of ACC deaminase and trehalose in *Pseudomonas* sp. UW4 in the protection of tomato plants against salt stress.

## Introduction

Salinity of agricultural soils is one of the main problems for farmers, since growth and plant production can be adversely affected. Soluble salts decrease the fertility of soils, producing osmotic stress, affecting water balance, and ion homeostasis. This alters the plant’s hormonal status, disturbing transpiration, nutrient acquisition and photosynthesis, among others (Munns and Tester, 2008; Ilangumaran and Smith, 2017). Plants contain various mechanisms to cope with salt stress, including a microbiome associated with the rhizosphere, phyllosphere, and endosphere (Orozco-Mosqueda et al., 2018). A group of organisms that stand out in this plant microbiome are the beneficial bacteria known as plant growth-promoting bacteria (PGPB). These bacteria, such as *Azospirillum, Arthrobacter, Azotobacter, Bacillus, Burkholderia, Enterobacter*, and *Pseudomonas*, have been shown to improve salt tolerance in several plant crops (Forni et al., 2017; Bharti and Barnawal, 2019).

Among the arsenal of mechanisms used by PGPB is the presence of the enzyme 1-aminociclopropane-1-carboxylase (ACC) deaminase, which converts ACC into ammonia and α-ketobutyrate. The ACC is a precursor of ethylene in all higher plants, and both ACC and ethylene typically increase in plants when they are under stress. Therefore, the ACC deaminase activity is one of the key traits used by PGPB to decrease ethylene levels under a wide range of stress conditions, including salinity (Mayak et al., 2004; Glick et al., 2007). Direct evidence for this mechanism comes from a study with *acdS* mutants of *Pseudomonas* sp. UW4 (ACC deaminase minus), which have lost the ability to promote the elongation of canola roots under gnotobiotic conditions (Li et al., 2000), and in presence of salt, are unable to promote growth of canola plants as the wild-type UW4 strain, which significantly improved plant growth (Cheng et al., 2007). The bacterium *Pseudomonas* sp. UW4 was isolated from the rhizosphere of common reeds and its genome is completely sequenced. In addition, the UW4 strain has been widely studied for its ability to promote plant growth under different environmental stresses, such as flooding, high concentrations of salt, cold, heavy metals, drought, and biocontrol of phytopathogens (Glick et al., 2007; Duan et al., 2013).

Another mechanism utilized by PGPB to reduce stress and promote growth in plants in the presence of both drought and high levels of salt is the production of trehalose (Suárez et al., 2008). Trehalose is a non-reducing disaccharide in which the two glucose units are linked via an α,α-(1,1)-glycosidic bond. This disaccharide has been isolated from all domains of life including plants, animals, fungi, yeast, archaea, and bacteria (Avonce et al., 2006; Chandra et al., 2011). Trehalose is also industrially produced as it is used in the food, cosmetics, and pharmaceutical industries (Lee et al., 2005; Wang et al., 2014). In addition, this disaccharide can play important and different roles in biological cells, such as, as a signaling molecule, reserve carbohydrate, and protectant against various stresses (e.g., drought, cold, and salt stress; Fernandez et al., 2010). Interestingly, the trehalose content has been strongly associated with the ability of some microorganisms to survive in harsh environments (Kandror et al., 2002; Cheng et al., 2007; Suárez et al., 2008).

Up to five different routes of trehalose biosynthesis have been described, most which include several steps (Paul et al., 2008; Fernandez et al., 2010). The only known route for trehalose synthesis in a single reaction step is the TreS pathway, where trehalose can be synthesized from maltose by trehalose synthase (TreS). Derived from the analysis of the genomic sequence of the strain UW4, it was reported that there may be two routes for the synthesis of trehalose in this bacterium, including treY/treZ, and treS (Duan et al., 2013). TreS interconverts maltose and alpha, alpha-trehalose by transglucosylation. This enzyme has also been found in a wide variety of bacterial species, such as *Azospirillum brasilense, Rhizobium etli, Corynebacterium glutamicum, Arthrobacter aurescens, Meiothermus ruber*, and *Pseudomonas stutzeri* (Avonce et al., 2006; Rodríguez-Salazar et al., 2009; Wang et al., 2014), consistent with its importance in survival under abiotic stress. In addition, a higher level of trehalose accumulation has been found in root nodules of *Medicago truncatula* and *Phaseolus vulgaris* in response to drought and salt stress, suggesting a key role of trehalose in signaling during plant-bacteria interactions by promoting plant growth, yield, and better adaptation to harsh conditions (López et al., 2008; Suárez et al., 2008). In the study reported herein, we constructed and tested a series of bacterial mutants in order to analyze the synergistic effects of ACC deaminase activity and trehalose accumulation on the ability of the plant growth-promoting bacterium *Pseudomonas* sp. UW4 to protect tomato (*Lycopersicon esculentum* cv. Saladette) plants against salt stress during growth.

## Materials and Methods

### Bacterial Strains and Media

Wild-type *Pseudomonas* sp. strain UW4 (Duan et al., 2013) was grown and maintained aerobically at 30°C in Nutrient Agar (NA) (BD Bioxon). The *Escherichia coli* strain was grown in Luria-Bertani (LB) medium (BD Bioxon) at 37°C. All strains were routinely maintained at 4°C. Antibiotics were added to the media when needed at the following concentrations (in μg ml^-1^): Carbenicillin (Cb), 100; Chloramphenicol (Cm), 20; Kanamycin (Km), 50; and Tetracycline (Tc), 10. For selection in cloning experiments, X-Gal (5-bromo-4-chloro-3-indolyl-D-galactoside) (Promega) was added to LB plates at 30 μg/ml.

### Construction of *Pseudomonas* sp. UW4 Mutants

The ACC deaminase minus mutant (*acdS^-^*) was previously constructed and characterized (Li et al., 2000). The wild-type *Pseudomonas* sp. UW4 strain was used to generate a mutation in the *treS* gene. First, a plasmid with the *treS* gene, interrupted by a chloramphenicol resistance cassette from pSUP5011 (in the single BamHI site of the *treS* sequence), was constructed (pJQ200SK/*treS*::Cm^r^) and transformed into the genome of the UW4 strain. Subsequently, clones were selected for a homologous double-crossover event that occurred between the wild-type *treS* gene and the disrupted *treS*::Cm^r^ gene on the replacement vector. The positive counterselection of clones with subsequent loss of the replacement vector was due to the presence of the *sacB* gene in the pJQ200SK, and therefore, sucrose sensitivity (Gay et al., 1985). In order to generate an *acdS^-^/treS^-^* double mutant, the aforementioned process was carried out with the exception that, instead of using the wild-type UW4 strain, the *acdS^-^* mutant was used. The double mutant was Tc^r^ and Cm^r^. In all cases, the mutants were checked for gene disruptions by PCR and DNA sequencing. To complement the *treS^-^* mutant, a broad-host-range cloning vector (pBBR1MCS-2) was used to carry the functional *treS* (pBBR1-*treS*) and express it under a constitutive promoter (derepressed P-lac) of the plasmid. Since the plasmid was a multicopy vector, the respective derivative strain over-expressed the *treS* gene and was named *OxtreS* strain. Procedures for genomic DNA isolation, plasmid isolation, gene cloning, and *E. coli* transformation were carried out using standard protocols (Sambrook and Russel, 2001). Table 1 shows all strains and plasmids used in this work.

**Table 1:** Bacterial strains and plasmids used in this work.

Strain/plasmid	Relevant phenotype	References or source
*Escherichia coli* DH5α	*SupE44*Δ*lacU169 (f80 lacZ*Δ*M15) hsdR17 recA1 endA1 gyrA96 thi-1 relA1, Nxr*	Life Technologies
*Escherichia coli* TOP10	*F^-^ mcrA*Δ*(mrr-hsdRMS-mcrBC) φ80lacZ*Δ*M15*Δ*lacX74 deoR recA1 araD139*Δ*(ara-leu)7697 galU galK rpsL endA1 nupG*	Invitrogen
pBluescriptII SK	*ColE1* mcs-*lacZ*, Ap^r^	Stratagene
pBBR1MCS-2	Km^r^	Kovach et al., 1995
pJQ200SK	Suicide vector with *lacZα* carrying *sacB*, Gm^r^	Quandt and Hynes, 1993
pSUP5011	Cm^r^	Simon et al., 1983
pBBR1-*treS*	pBBR1MCS-2 containing *treS* gene	This work
pJQ200SK/*treS*::Cm^r^	pJQ200SK containing *treS*::Cm^r^	This work
*Pseudomonas* sp. UW4	Wild-type	Duan et al., 2013
UW4 *acdS^-^*	*acdS*::Tc^r^	Li et al., 2000
UW4 *treS^-^*	*treS*::Cm^r^	This work
UW4 *acdS^-^/treS^-^*	*acdS*::Tc^r^/*treS*::Cm^r^	This work
UW4 *OxtreS*	*treS* overexpressing, cloned in pBBR1MCS-2	This work


### Survival Experiments of Bacterial Strains on Salt Stress

Survival of *Pseudomonas* sp. strain UW4, mutants (*acdS, treS*, and *acdS/treS*), and the trehalose over-expressing strain (*OxtreS*) derivative under conditions of salt stress (NaCl) was measured after 48 h of growth on minimal medium (M9) (Sigma), as colony forming units per ml. A pre-inoculum with 1.0 × 10^6^ (± 0.01 × 10^6^) of CFU ml^-1^ was used for each strain. The minimal medium was supplemented with 0, 0.2, or 0.8 M NaCl, and cultures were incubated with shaking for 48 h at 30°C.

### Determination of ACC Deaminase Activity

Plant growth-promoting bacteria containing the ACC deaminase enzyme are able to use ACC as a sole nitrogen source. Therefore, the determination of the ACC deaminase activity in the strains generated in this work was evaluated as previously described by Penrose and Glick (2003). This method quantifies the amount of α-ketobutyrate produced as a result of the cleavage of ACC by ACC deaminase. The final ACC deaminase activity was expressed in μmol α-ketobutyrate mg protein^-1^ h^-1^. Wild-type strain *Pseudomonas* sp. UW4 was used as a positive control in all replicates.

### Quantification of IAA Production

The determination of indole-3-acetic acid (IAA) production in bacterial strains used in this work was performed by Gas Chromatography and Mass Spectrometry, as previously reported by Hernández-León et al. (2015). Nevertheless, some modifications for specific estimation in bacteria were applied. The identity of IAA was confirmed by comparison of the retention time in the bacterial extracts with samples of the pure IAA standard (Sigma). For estimation of the IAA amount produced by strains, an individual calibration curve was constructed. The IAA determinations were done in triplicate.

### Trehalose Quantification

The accumulation of the disaccharide trehalose in wild-type UW4, the isogenic mutants, and the trehalose overexpressing strain was measured by high performance liquid chromatography (HPLC). Briefly, cell cultures of 250 ml were prepared in minimal medium (M9) with 4% glycerol and the culture was left for 48 h under agitation at 30°C. The medium supernatant was filtered (MF-Millipore membrane filters 0.22 μm) and 1.5 ml of sterile deionized water was added. Trehalose concentration was determined by HPLC with a carbohydrate analysis column Aminex HPX-87C (Bio-Rad Labs, Richmond, CA, United States). A standard of pure trehalose (Sigma) was employed as a reference to identify the peak of the disaccharide.

### Evaluation of Plant Growth-Promotion in the Presence of Salt Stress

Greenhouse experiments with tomato plants (*L. esculentum* cv. Saladette) were performed according to the methods described by Rojas-Solís et al. (2018). The experiments were carried out in pots (6 cm tall × 5 cm wide) with sterile peat moss (Sphaigne, Canada), with or without irrigation with a salt solution (0.2 M NaCl). Previous screening experiments with different salt stresses (0, 0.1, 0.2, 0.4, and 0.8 M NaCl) demonstrated that a concentration of 0.2 M of NaCl was considered a saline stress for tomato plants after comparing the growth and chlorophyll parameters of these plants with control plants devoid of salt stress. Subsequently, tomato seeds were germinated *in vitro*, and after 1 week, seedlings of the same size were selected and transplanted into pots (one plant per pot). Bacterial inoculants dissolved in sterile deionized water were applied every week after pot transplantation according to the experimental design, which also included treatments without bacterial inoculations. The concentration of bacterial inoculants was adjusted such that their optical density at 600 nm was 1 (∼0.75–1 × 10^8^). The plants were irrigated every 3 days with deionized water throughout the whole experiment.

Each of the experimental treatments (Control, Control+NaCl, Control+each of the 5 strains, and Control+NaCl+each of the 5 strains) included 12 plants. Since the experiment was repeated twice, the total experimental units were 288. The effect of each of the bacterial inoculants on the root length, aerial parts, total dry weight, and chlorophyll concentration was evaluated after 5 weeks of plant growth. The chlorophyll concentration was measured in three leaves from each plant, as previously reported by Rojas-Solís et al. (2018).

### Statistical Analysis

As mentioned above, plant growth promoting experiments were performed twice. The results were analyzed using Statistica 6.0 software (StatSoft Inc., 2001). The Student’s *t-*test was used to compare the means of two groups, and the ANOVA and Duncan’s means separation test were used for multiple comparisons (*p* < 0.05).

## Results

### Survival of Wild-Type and Mutants on Salt Stress

Survival of the wild-type strain *Pseudomonas* sp. UW4, the isogenic mutants (*acdS, treS*, and *acdS/treS*), and the trehalose over-expressing strain (*OxtreS*) derivative were evaluated in two salt concentrations: 0.2 and 0.8 M NaCl (Table 2). The results show that, in all strains, very little significant (*p* < 0.05) decrease in the survival was observed at 0.2 M NaCl. However, at 0.8 M, a decrease in the survival of all strains was noted. It should be noted that in mutants *treS* and *acdS/treS*, the decrease in survival was more marked, and a four- to fivefold decrease in survival relative to the wild-type was observed.

**Table 2:** Survival of *Pseudomonas* sp. strain UW4, mutants, and overexpressing strain derivatives.

UW4 derivative strains	Survival without saline stress (0 M NaCl)	Survival on saline stress (0.2 M NaCl)	Survival on saline stress (0.8 M NaCl)
Wild-type	2.2 × 10^8^ ( ± 0.23 × 10^8^) a	1.2 × 10^8^ ( ± 0.16 × 10^8^) a	2.8 × 10^6^ ( ± 0.39 × 10^6^) b
*acdS^-^*	1.1 × 10^8^ ( ± 0.18 × 10^8^) a	0.8 × 10^8^ ( ± 0.68 × 10^8^) a	4.5 × 10^4^ ( ± 0.24 × 10^4^) c
*treS^-^*	0.9 × 10^7^ ( ± 0.77 × 10^7^) b	0.5 × 10^7^ ( ± 0.22 × 10^7^) b	1.8 × 10^3^ ( ± 0.54 × 10^3^) c
*acdS^-^/treS^-^*	0.2 × 10^7^ ( ± 0.48 × 10^7^) b	0.1 × 10^7^ ( ± 0.21 × 10^7^) b	3.8 × 10^2^ ( ± 0.39 × 10^2^) d
*OxtreS*	3.2 × 10^8^ ( ± 0.13 × 10^8^) a	1.0 × 10^8^ ( ± 0.13 × 10^8^) a	2.1 × 10^7^ ( ± 0.23 × 10^7^) b


*Survival was measured after 48 h of growth in minimal media as colony forming units per ml; pre-inoculum of each strain contained 1.0 × 10^*6*^ ( ± 0.01 × 10^*6*^) of CFU ml^-*1*^. The standard deviation is shown in parentheses (±). Different letters indicate significant differences at α = 0.05.*

### Analysis of ACC Deaminase Activity and IAA Synthesis

**FIGURE 1:**
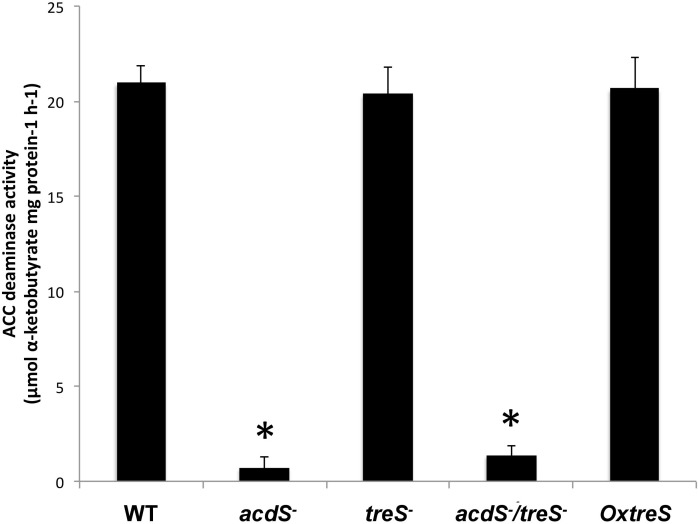
Evaluation of ACC (1-aminocyclopropane-1-carboxylate) deaminase activity in wild-type (WT) bacterium *Pseudomonas* sp. UW4; its derivative mutant strains in the *acdS, treS*, and *acdS*/*treS* genes; and the overexpressing *OxtreS* (Trehalose synthase) strain. The figure represents the results of at least three independent experiments. Asterisks indicate that the means of the samples are different from the WT at *P* < 0.05.

The ACC deaminase activity was analyzed either in strain UW4 or the ACC deaminase minus mutant. The wild-type strain showed the ability to use ACC as a sole source of nitrogen, while the *adcS^-^* strain showed no ACC deaminase activity (Figure 1). The mutant strains *treS* and *OxtreS* did not show any change in ACC deaminase activity, while the double mutant *acdS/treS* did not show any ACC deaminase activity.

When monitoring the production of indoleacetic acid in the analyzed mutants, no significant changes were observed compared to the wild-type strain of *Pseudomonas* sp. UW4. The results of this analysis are shown in Table 3.

**Table 3:** Indole acetic acid (IAA) production of *Pseudomonas* sp. UW4 and derivative strains.

UW4 derivative strains	IAA production (μg/ml)
Wild-type	3.13 ± 0.6
*acdS^-^*	2.92 ± 0.4
*treS^-^*	3.08 ± 0.5
*acdS^-^/treS^-^*	2.55 ± 0.9
*OxtreS*	2.89 ± 0.7


*The standard deviation is shown in parentheses (±). Statistical analysis indicated no significant differences at α = 0.05.*

### Production of Trehalose

**FIGURE 2:**
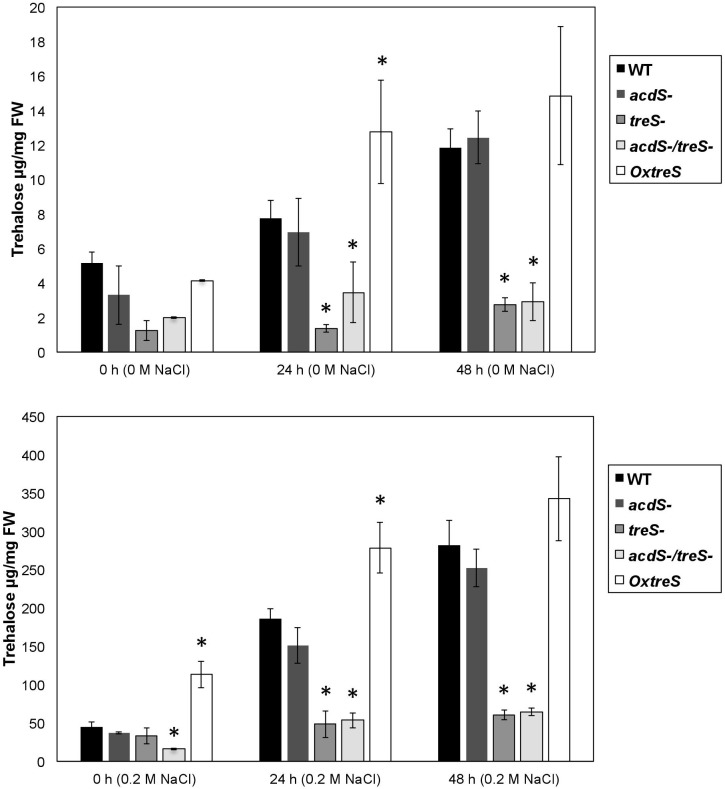
Analysis of trehalose accumulation in wild-type (WT) Pseudomonas sp. UW4; its derivative mutants *acdS, treS*, and *acdS*/*treS*; and the overexpressing *OxtreS* (*OxtreS*) strain without **(Upper panel)** and with salt **(Lower panel)**. The figures exhibit the results of at least three independent experiments. Asterisks indicate that the means of the samples are different from the WT at *P* < 0.05.

The accumulation of trehalose after 0, 24, and 48 h without salt conditions can be observed in Figure 2A. Specifically, the wild-type and the ACC deaminase minus mutant strains increased trehalose production at 24 and 48 h of growth, while *OxtreS* showed an even greater accumulation than the other strains, even in unsalted media. On the other hand, the *treS* and *acdS/treS* mutants significantly decreased their accumulation of trehalose compared with the strains not affected in the *treS* gene.

In Figure 2B, the quantification of trehalose is observed in growth media supplemented with 0.2 M NaCl. It should be noted that the accumulation of trehalose in the overexpressing strain was greater than that in the other strains, including the wild-type strain, which increased its accumulation after 24 and 48 h. The *treS* and *acdS/treS* mutants also exhibited a slight increase in the accumulation of trehalose in the presence of salt; however, this increase was less marked than in the wild-type strain. This suggests that there are other routes for the production of trehalose in *Pseudomonas* sp. UW4; nevertheless, the mutation in the *treS* gene significantly reduced the production of trehalose under high-salinity conditions, which correlates with a lower survival of bacteria that contained this mutation in media with NaCl (Table 2).

### Tomato Growth-Promotion on Salt Stress

**FIGURE 3:**
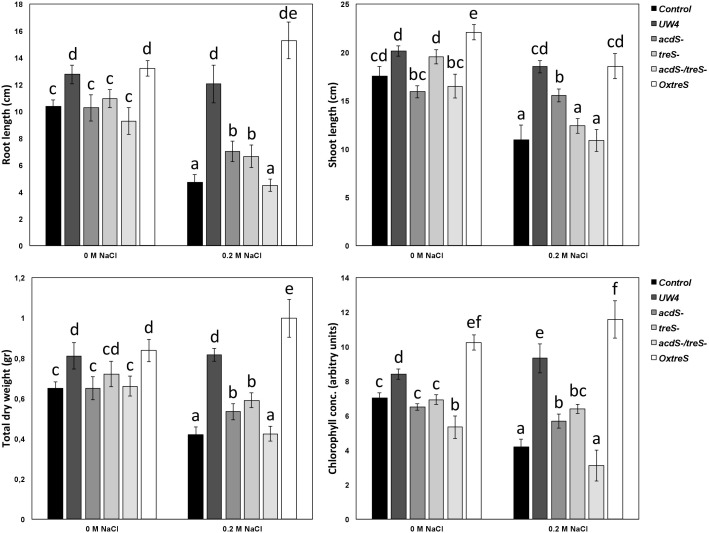
Evaluation of tomato (*Lycopersicon esculentum* cv Saladette) growth promoting effects by the inoculation of *Pseudomonas* sp. UW4 WT, single *acdS* and *treS* mutants, double mutant *acdS*/*treS*, and the overexpressing *OxtreS* (TreS) strain. Bars represent the mean ± SE values (*n* = 12). Different letters indicate significant differences (*p* < 0.05; Duncan’s multiple range test).

**FIGURE 4:**
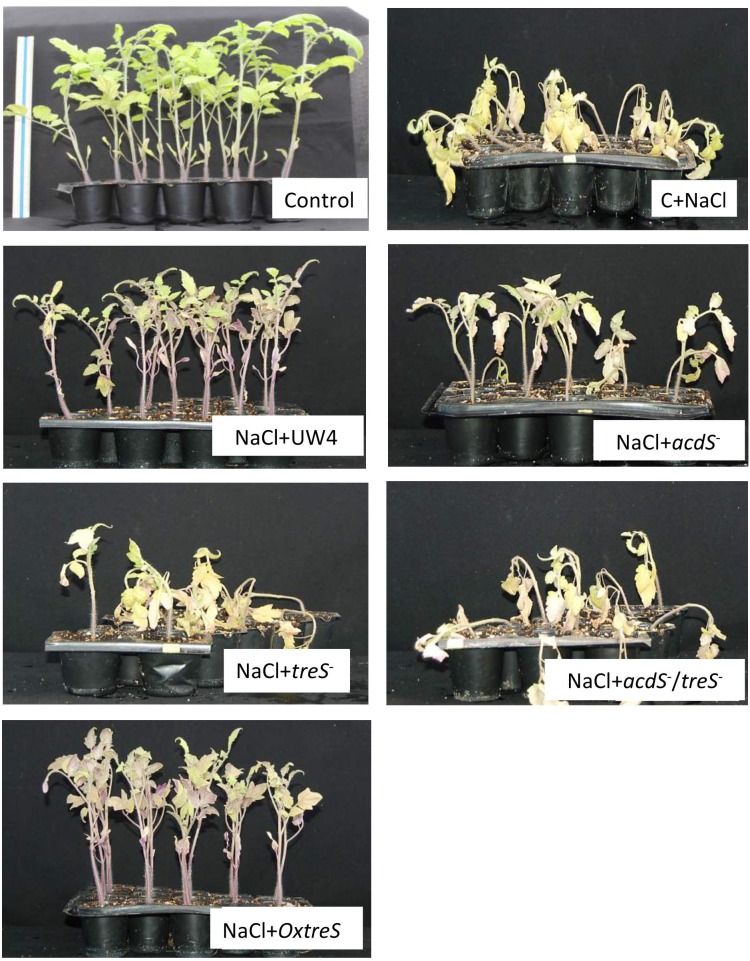
Composite picture of tomato (*L. esculentum* cv *Saladette*) plants inoculated with the indicated bacterial strains. Control plants contained no salt or bacteria. C+NaCl (0.2 M) indicates plants irrigated with salt. See the Section “Materials and Methods” for details of the experiment. The rest of the panels show plants + salt (0.2 M) + each of the strains (WT *Pseudomonas* sp. UW4, *acdS^-^, treS^-^, acdS^-^* /*treS^-^*, and *OxtreS*).

Bacterial ACC deaminase activity and trehalose production has been evaluated (separately) in several studies (Li et al., 2000; Cheng et al., 2007; Suárez et al., 2008). However, in this study, the synergy of both processes in promoting tomato plant growth was evaluated using plant the growth-promoting strain *Pseudomonas* sp. UW4 and its different mutants in pot experiments. The wild-type strain UW4 maintained its growth-promoting capacities under conditions of salt stress in tomato plants by increasing plant root and shoot length, total dry weight, and chlorophyll content to levels similar to those in plants that were not subjected to NaCl stress (Figure 3). The *acdS* and *treS* mutants showed a similar trend (to one another) when used to inoculate plants under salt stress, failing to improve the parameters evaluated in tomato plants. Surprisingly, plants inoculated with the double mutant showed a beneficial effect in three parameters, namely root length, total dry weight, and chlorophyll content, showing similar values to control plants subjected to uninoculated saline stress. In addition, inoculation of plants with the double mutant resulted in lower values than with plants inoculated with each of the single *acdS* and *treS* mutants. Finally, the inoculation of the *OxtreS* strain showed a significant improvement in root length (greater than the wild-type UW4 strain), whereas for the other evaluated parameters, it showed beneficial effects that were statistically similar to those observed for the wildtype UW4 strain (Figure 4).

## Discussion

Organisms have evolved different mechanisms to counteract the damaging effects of different adverse environmental conditions, such as soil salinity, which is an enormous problem in agriculture. During salt stress, plants increase their ethylene synthesis, thereby inhibiting their growth and productivity (Glick et al., 2007). One strategy to reduce ethylene levels in plants has been the inoculation of plants with bacteria that contain ACC deaminase activity. Thus, it has been observed that bacteria of different genera, such as *Achromobacter, Azospirillum, Brevibacterium, Bacillus*, or *Pseudomonas*, can mitigate the toxic effects of high-salinity, increasing biomass, chlorophyll content, and production (Bacilio et al., 2004; Mayak et al., 2004; Cheng et al., 2007; Siddikee et al., 2011; Ali et al., 2014). However, not all bacteria that help mitigate the toxic effects of salt on plants contain the genetic machinery essential for ACC deaminase activity (Blaha et al., 2006; Duan et al., 2009; Ramadoss et al., 2013); other beneficial bacterial mechanisms can help to mitigate the inhibitory effects of high salt. For example, PGPB that produce IAA under saline conditions may supply an additional amount of this phytohormone to plants. The supplementary IAA may help to stimulate root growth and partially reverse the growth inhibiting effects of salt stress in both shoot and root growth, as well as improving other physiological parameters like chlorophyll content (Duca et al., 2018). In this regard, the mutants constructed here did not show any impairment in IAA synthesis, since all of them showed similar production when compared to the wild-type strain (Table 3). Therefore, the beneficial role of IAA (or other compounds) still produced by the mutants cannot be excluded from the low level of beneficial effects observed in tomato plants during inoculation.

Another relevant and widely studied mechanism of how bacteria facilitate plant growth during salt and drought stress is the production of metabolites such as trehalose (Avonce et al., 2006). Trehalose is a non-reducing disaccharide that is widely present in different groups of organisms and plays diverse roles such as an energy source molecule, in addition to being important during osmotic, heat, and desiccation stress tolerance (Iordachescu and Imai, 2008). Moreover, trehalose synthesis is also triggered by salinity stress in many rhizobacteria (Suárez et al., 2008) and the importance of trehalose in the osmostress response has been well established in several rhizobia (Domínguez-Ferreras et al., 2009; Sugawara et al., 2010; Reina-Bueno et al., 2012).

In this work, using tomato as a model plant, we tested the hypothesis that the ACC deaminase activity of *Pseudomonas* sp. UW4 and the production of trehalose by this bacterium play a synergistic role in protecting plants from the growth inhibitory effects of high salinity. The results of this study first confirmed that the mutant of strain UW4 that does not contain ACC deaminase activity does not exhibit growth-promoting effects on tomato, consistent with the results observed in other studies, where ACC deaminase minus mutants did not confer resistance to salt stress in canola (Li et al., 2000; Cheng et al., 2007) or tomato (Ali et al., 2014).

Some bacteria contain up to five disaccharide biosynthesis pathways (Avonce et al., 2006; Paul et al., 2008). Therefore, a first mutation was generated in the *treS* gene of *Pseudomonas* sp. UW4. When analyzing the accumulation of trehalose in this mutant, it was observed that a large percentage of the total accumulated trehalose was decreased (Figure 3). As mentioned earlier, there appear to be two routes of synthesis of trehalose in the bacterium *Pseudomonas* sp. UW4, so the *treS* pathway would be the most important for the production of trehalose in this strain (Duan et al., 2013), although it would be necessary to generate other mutants to determine the validity of such a hypothesis. This result also agrees with previous work showing that there are multiple routes of trehalose synthesis and its synthesis is not completely abolished by abolishing a single pathway (Suárez et al., 2008). Nevertheless, the mutation in *treS* affected the beneficial effect of the strain UW4 when it was used to inoculate tomato plants, since, compared to the control plants inoculated with the wild strain, significant decreases in the length of the tomato plant roots and shoots, chlorophyll concentration, and total dry weight were observed. This suggests that the TreS pathway in strain UW4 plays a key role in the production of trehalose and in subsequently mitigating the toxic effects of salinity in tomato plants. Other studies have shown the importance of this route in bacteria such as *Mycobacterium smegmatis* (Pan et al., 2004). Also, in a recent work, a new *treS* gene (TreS) derived from a metagenomic analysis of a saline-alkaline soil was identified (Jiang et al., 2013). The protein (TreS) was characterized as follows: it showed good production of trehalose from maltose, was stable, and possessed different biochemical characteristics compared to other TreSs (Jiang et al., 2013). The *treS* gene from *Pseudomonas putida* KT2440 has also exhibited good potential for trehalose production (Wang et al., 2014). Streeter and Gomez (2006) analyzed three trehalose biosynthetic pathways in *Bradyrhizobium*, either in a free-living state or during symbiosis with plants. Interestingly, these authors observed that TreS was the dominant enzyme in bacteroids, although the substrate for TreS, maltose, was present in very low concentration in nodules. The precise role(s) of the alternative routes of trehalose synthesis in bacteria and how they affect plant-bacteria interactions remains to be elaborated.

To test the hypothesis that bacterial ACC deaminase activity and trehalose accumulation act synergistically in the protection of plants against toxic saline effects, a double mutant, *acdS/treS Pseudomonas* sp. UW4, was constructed. The results show that the double mutant was significantly impaired in the beneficial effects of the wild-type bacterium on the length of tomato plant roots, total plant dry weight, and chlorophyll content, in the presence of salt, and in general, these plants behaved similarly to control (uninoculated) plants subjected to saline stress. It is worth mentioning that inoculation with each of the single *acdS* or *treS* mutants showed better protection against saline conditions than plants inoculated with the double mutant *acdS/treS*, suggesting a synergistic role of these traits.

Stearns et al. (2012) analyzed the genetic response of *Arabidopsis* to the inoculation of ACC deaminase positive and negative strains of *Pseudomonas* sp. UW4. The microarray results from those experiments demonstrated that the transcription of genes involved in plant hormone regulation, stress response, and secondary metabolism was modified in plants by the presence of the bacterial strains, whereas the upregulation of genes for auxin response factors and the downregulation of stress response genes was observed only in the presence of the ACC deaminase positive strain. In those experiments, the trehalose biosynthetic genes (TPS/TPP) in plants were weakly downregulated (-1.7 to -1.8-fold change) by the inoculation of both ACC deaminase positive and negative strains, suggesting a link between the bacterium and the plant’s trehalose metabolism, but not a direct link to the ACC deaminase activity. It would be interesting to further evaluate the mutants of *Pseudomonas* sp. UW4 generated in the present study (*treS, acdS/treS*, and *OxtreS*) in relation to the gene expression and metabolism of plants in order to explore possible novel signaling pathways in plant-bacteria interactions.

In the present work, a second strain with the *treS* gene was constructed to overproduce RNA copies of the *treS* gene (*OxtreS* strain). The *OxtreS* strain significantly overproduced trehalose compared to the wild-type stain, growing either in media supplemented or not with NaCl. Trehalose overaccumulation was most evident after 24 h of growth. After 48 h of growth, trehalose accumulation in the *OxtreS* strain was only slightly (not significantly) higher than in the wild-type strain (UW4). It is worth mentioning here that wild-type UW4 showed good accumulation of trehalose during growth after 24 and 48 h in media with 0.2 M of NaCl. When plants were inoculated with the *OxtreS* trehalose overexpressing strain, it was notable that the resultant plants displayed improved root length, dry weight, and chlorophyll content when compared with the wild-type strain. These results agree with other studies where the previously mentioned strategies protect plants of agronomic interest under conditions of stress (Orozco-Mosqueda et al., 2018). In fact, it has been proposed that the physiological responses of plants to salinity are similar to other environmental stresses such as drought, and that therefore, they may share common stress-tolerance pathways (Munns and Tester, 2008; Forni et al., 2017). For example, the overexpression of trehalose-6-phosphate synthase gene (*otsA*) has been analyzed in *Rhizobium etli*, where the modified strain improved drought tolerance and grain yield in *P. vulgaris* (Suárez et al., 2008). Similarly, a genetically engineered strain of a plant growth-promoting strain of *Azospirillum* with an improved level of trehalose biosynthesis increased both drought tolerance and plant biomass in maize plants (Rodríguez-Salazar et al., 2009).

In conclusion, this work demonstrated that ACC deaminase and trehalose synergistically protect tomato plants against salt stress during the interaction with the PGPB *Pseudomonas* sp. UW4. It remains to be elucidated whether the inoculation of other plants with these mutants affects plants other than tomato in a similar manner and in particular whether appropriately engineered bacteria might facilitate grain or fruit production of plant growing under different stresses.

## Data Availability

All datasets generated for this study are included in the manuscript and/or the Supplementary Files.

## Author Contributions

MO-M conducted the experiments, analyzed the data, and prepared the figures and/or tables. JD, MD, and EZ conducted the experiments and prepared the figures and/or tables. JC-G analyzed the data and reviewed drafts of the manuscript. BG conceived and designed the experiments, analyzed the data, and reviewed drafts of the manuscript, and approved the final draft. GS conceived and designed the experiments, wrote the manuscript, and approved the final draft. All authors read and approved the final version of the manuscript.

## Conflict of Interest Statement

The authors declare that the research was conducted in the absence of any commercial or financial relationships that could be construed as a potential conflict of interest.
